# New Starch Phenotypes Produced by TILLING in Barley

**DOI:** 10.1371/journal.pone.0107779

**Published:** 2014-10-01

**Authors:** Francesca Sparla, Giuseppe Falini, Ermelinda Botticella, Claudia Pirone, Valentina Talamè, Riccardo Bovina, Silvio Salvi, Roberto Tuberosa, Francesco Sestili, Paolo Trost

**Affiliations:** 1 Department of Pharmacy and Biotechnology FABIT, University of Bologna, Bologna, Italy; 2 Department of Chemistry “G. Ciamician”, University of Bologna, Bologna, Italy; 3 Department of Agriculture, Forestry, Nature & Energy, University of Tuscia, Viterbo, Italy; 4 Department of Agricultural Sciences, University of Bologna, Bologna, Italy; University of Potsdam, Germany

## Abstract

Barley grain starch is formed by amylose and amylopectin in a 1∶3 ratio, and is packed into granules of different dimensions. The distribution of granule dimension is bimodal, with a majority of small spherical B-granules and a smaller amount of large discoidal A-granules containing the majority of the starch. Starch granules are semi-crystalline structures with characteristic X-ray diffraction patterns. Distinct features of starch granules are controlled by different enzymes and are relevant for nutritional value or industrial applications. Here, the Targeting-Induced Local Lesions IN Genomes (TILLING) approach was applied on the barley TILLMore TILLING population to identify 29 new alleles in five genes related to starch metabolism known to be expressed in the endosperm during grain filling: *BMY1* (*Beta-amylase 1*), *GBSSI* (*Granule Bound Starch Synthase I*), *LDA1* (*Limit Dextrinase 1*), *SSI* (*Starch Synthase I*), *SSIIa* (*Starch Synthase IIa*). Reserve starch of nine M3 mutant lines carrying missense or nonsense mutations was analysed for granule size, crystallinity and amylose/amylopectin content. Seven mutant lines presented starches with different features in respect to the wild-type: (i) a mutant line with a missense mutation in *GBSSI* showed a 4-fold reduced amylose/amylopectin ratio; (ii) a missense mutations in *SSI* resulted in 2-fold increase in A:B granule ratio; (iii) a nonsense mutation in *SSIIa* was associated with shrunken seeds with a 2-fold increased amylose/amylopectin ratio and different type of crystal packing in the granule; (iv) the remaining four missense mutations suggested a role of *LDA1* in granule initiation, and of *SSIIa* in determining the size of A-granules. We demonstrate the feasibility of the TILLING approach to identify new alleles in genes related to starch metabolism in barley. Based on their novel physicochemical properties, some of the identified new mutations may have nutritional and/or industrial applications.

## Introduction

Barley (*Hordeum vulgare* L.) is the fourth most important cereal crop both in terms of cultivated area and tonnage harvested; global production being mostly used as animal feed and for the malting industry (http://faostat.fao.org). Only 5% of the global production of barley is used as ingredients in food preparation, but nevertheless barley grains are a valuable functional food for the high content of soluble dietary fiber [1]. The recent assemblage of the sequence of the 5.1-Gb haploid genome of barley [2] further supports the role of barley as a model species for the Triticeae tribe, which includes very important crops such as wheat (bread and durum) and rye.

Mature barley grains typically contain 50–60% starch on a dry weight basis. Starch is synthesized and stored in granules composed of two types of D-glucose polymers, amylose and amylopectin. Amylose, generally accounting for about 25–30% of starch weight in barley, is essentially a linear polymer of D-glucose units linked by alpha-1,4-glucosidic bonds. The second polymer of starch, amylopectin, is highly branched because of the alpha-1,6-glucosidic bonds that connect short alpha-1,4 linear chains [3], [4], [5], [6], [7], [8], [9].

While most plants contain starch granules of similar size, the Triticeae endosperm presents two classes of starch granules characterized by different sizes and shapes [10], [11]. Most of the starch is stored in large A-granules, but small B-granules prevail in number. In barley, the diameter of A-granules ranges from 10 to 40 µm while B-granules are smaller than 10 µm [10]. Starch granules contain crystalline lamellae in which double helices, composed of parallel linear chains of amylopectin, interact among each other to form different types of crystal packing [3]. Crystalline lamellae are interspersed with amorphous lamellae in which amylopectin branches are concentrated. The exact location of amylose within the semicrystalline architecture of the starch granule is still unknown [9], but certainly amylose influences the global structure of starch granules. For example, starch granules of different composition are characterized by different types of X-ray diffraction patterns [3], [4], [8]. In cereals, the A-type crystal packing is predominant, while the B-type crystallinity, typical of tuber starch, exists in smaller amounts. A third diffraction pattern, named V-type, is associated with lipid-amylose complexes and is little represented in native starches [3].

Both amylose/amylopectin ratios and the architecture of starch granules depend in a complex way on many different enzymes involved in starch metabolism [4], [5], [8], [9]. Biosynthesis of starch polymers in cereal grains strictly depends on the availability of ADP-glucose as a precursor for both amylose and amylopectin polymerization. Starting from ADP-glucose, a single enzyme, the granule-bound starch synthase I (GBSSI), is required for the synthesis of amylose. More complex is the biosynthetic pathway leading to amylopectin production as different classes of soluble starch synthases (SSs) and starch branching and debranching enzymes are required [5], [8], [9].

Starches with different amylose/amylopectin ratios have different properties that influence their possible use for either nutritional purposes or industrial transformations [6], [7], [9], [12], [13], [14]. In barley, mutants with 0–10% amylose (*waxy*) as well as mutants containing up to 70% amylose in the endosperm have been described [15], [16], [17]. Low-amylose starch display higher freeze-thaw stability, an interesting property for food preparation [6]. On the other hand, high amylose starches have interesting nutritional properties due to their positive correlation with resistant starch. This starch fraction is highly resistant to human digestion in the small intestine and reaches the large bowel where it plays a role similar to dietary fiber. Consumption of high-amylose resistant starch is associated with several health benefits, including the prevention of colon cancer, type II diabetes, obesity and cardiovascular diseases [18], [19].

Starch granule size distribution is another important parameter that may affect technological properties and end-use of each particular type of starch [20]. Barley grains are largely used for malting and large A-granules are more readily attached by hydrolytic enzymes than small B-granules [10]. B-granules are apparently protected during malting by a heterogeneous matrix deriving from the grain (proteins and cell walls). As a result, a significant proportion of B-granules escapes degradation and causes several technological problems during beer production [21].

In a previous work using a TILLING strategy, novel allelic variants in genes involved in starch metabolism in barley seeds were identified [22]. Here we describe the starch phenotype of nine mutants carrying either missense or nonsense mutations in five starch-related genes known to be expressed in the endosperm during grain filling: *BMY1* (beta-amylase 1), *GBSSI* (Granule Bound Starch Synthase I), *LDA1* (Limit Dextrinase 1), *SSI* (Starch Synthase I), *SSIIa* (Starch Synthase IIa). Seven mutant lines present starches with potentially interesting features for nutritional uses and/or industrial applications, including an altered amylose/amylopectin ratio or an unusually high percentage of A-granules or A-granules that are larger than in wild type starch.

## Materials and Methods

### TILLING analysis and plant materials

Details on the TILLING-based molecular screening for the five starch metabolism enzymes were reported in [22] and will only be summarized here. TILLMore is a chemically (sodium-azide, NaN_3_) mutagenized barley population including 4,906 M_3∶4_ families [23]. TILLMore was screened using a standard TILLING protocol based on LI-COR vertical gel electrophoresis of PCR reactions obtained on 8X bulked genomic DNA samples. Genes tilled were *Beta-amylase 1* (*BMY1*), *Granule-Bound Starch Synthase I* (*GBSSI*), *Limit dextrinase 1* (*LDA1*), *Starch Synthase I* (*SSI*) and *Starch Synthase IIa* (*SSIIa*) (Table 1). For each mutant, plant materials phenotyped in this work were grains (kernels) of M_4_ lines (three generations of selfing after mutation induction), which have been verified to be homozygous for the mutation (not shown). Mutant lines and cv. Morex were grown in open field following standard agronomic practice in 0.5-m long one-row plots (approx. 12 plants per plot) using a randomized design with two replicates. For each line, grains harvested (from all well-grown ears) from two replicates were bulked. The same experiment was carried out in two years. Grains from separated years constituted the biological replicates.

**Table 1:** List of TILLING mutant lines carrying either missense or nonsense mutations in five genes related to starch metabolism in barley grains that have been isolated as described in Bovina et al. [22] and phenotypically characterized in this work.

Gene name	Mutant code	Genebank accession	Nucleotide change	Amino acid substitution	Seed and starch phenotype
Beta-amylase 1	2253-*BMY1*	EF175470	G2522A	D277N	Normal
	2682-*BMY1*	EF175470	G2944A	E348K	Normal
Granule-bound starch synthase I	1090-*GBSSI*	AB088761	G2306A	G493E	Low % amylose. Low % V-diffraction pattern
Limit dextrinase 1	905-*LDA1*	AF122050	G1528A	V270I	Low % A- granules
Starch synthase I	1132-*SSI*	AF234163	C5705T	T522I	High % A- granules
	1284-*SSI*	AF234163	G5666A	G509E	Low % A- granules. Large A-granules
	5850-*SSI*	AF234163	G6020A	G576D	High % A- granules. Small A-granules
Starch synthase IIa	1039-*SSIIa*	AY133251	G2453A	G678R	Small A-granules
	1517-*SSIIa*	AY133251	G2449A	W676*	Small and shrunken seeds. Low % total starch. High % amylose. High % V-diffraction pattern. Deformed granules

### Starch extraction from barley grains

Starch was extracted by grinding the grains to a fine powder in a pepper mill. About 5 g of seeds, corresponding to about 100 seeds, were used for each line and for each biological replicate (except for line 1517-*SSIIa* for which 2.5 g of seeds were used). Starch grains were purified as described in [24]. Briefly, the powder was suspended in 70 ml Extraction Buffer (EB: 55 mM Tris-HCl, pH 6.8, 2.6% SDS, 10% glycerol, 2% ß-mercaptoethanol) and vigorously shaken for 48 h, replacing the EB solution every 24 h. Following the extraction, samples were washed three times in water and filtered through a nylon membrane (cut-off 100 µm) in order to eliminate debris. Filtered samples were spun down for 1 min at 10,000 g. Starch grains were resuspended in 25 ml acetone and spun down again. Once removed the supernatant, starch grains were air-dried under a chemical hood for about 48 h at room temperature.

### SDS-PAGE analysis of starch granule proteins

Isolation and electrophoretic separation of starch granule proteins was carried out on mature seeds following the method reported by Zhao and Sharp [25] with some modifications, as reported by Mohammadkhani et al. [26]. Protein bands were visualized by silver staining.

### Determination of total starch and amylose content

Total starch content was determined on whole flours using Megazyme Total Starch Assay Kit (Megazyme, Ireland). The relative content of amylose was determined using both the Amylose/Amylopectin assay kit (Megazyme, Ireland) following the manufacturer instructions, and by an iodometric assay as reported in Sestili et al. [27]. Three technical replicas were performed for each mutant and each type of measure. Total starch content and relative amylose content (enzymatic method) were measured on two biological replicas.

### Starch morphology

The morphology of starch samples was analyzed using a scanning electron microscopy (SEM) Hitachi S-4000. The samples were glued by a carbon type on an aluminum stub and gold coated (2 nm thick layer) before observations. For each sample two sets of 10 pictures at two magnifications (1000x and 2500x) were randomly collected. These two magnifications allowed a good estimation of the size of large and small granules. The granule size was estimated using the software ImageJ for image processing and analysis. The two main axes for each granule were recorded and 200–500 grains were measured for each sample. Percentage of granules with major axis lower than 8 µm (B-granules) was recorded in 10 pairs of pictures (at different magnification) for each genotype. Statistically significant differences between mutants and wild type mean values were detected by Student's t-test (P<0.01). The frequency analysis was carried out tacking classes of 2 µm.

### Starch crystallinity

The X-ray powder diffraction patterns were recorded using a Philips X'Celerator diffractometer with Cu Ka radiation (l = 1.5418 Å) and equipped with a Ni filter. The samples were scanned for 2θ angles between 5° and 30°, with a resolution of 0.02°. The degree of crystallinity of samples was quantitatively estimated following the method of Nara and Komiya [28]. A smooth curve which connected peak baselines was computer-plotted on the diffraction patterns. The area above the smooth curve was taken to correspond to the crystalline portion, and the lower area between the smooth curve and a linear baseline which connected the two points of intensity at 2θ of 30° and 5°. The upper diffraction peak area and total diffraction area over the diffraction angle 5°–30° 2θ were integrated on X'Pert HighScore Plus software (PANalytical B.V. 2008). The ratio of upper area to total diffraction area was taken as the degree of crystallinity.

In the diffraction patterns only peaks associable to A-type and V-type crystallinities were detected. To estimate the relative amount of A-type and V-type crystallinities in the starch samples from the diffraction patterns, only well isolated diffraction peaks were considered. The one at 15.1° is diagnostic of the A-type crystallinity and the one at 19.7° is diagnostic of the V-type one. These diffraction intensities has been normalized on the sum of their intensities.

## Results

### TILLING molecular analysis

**Figure 1:**
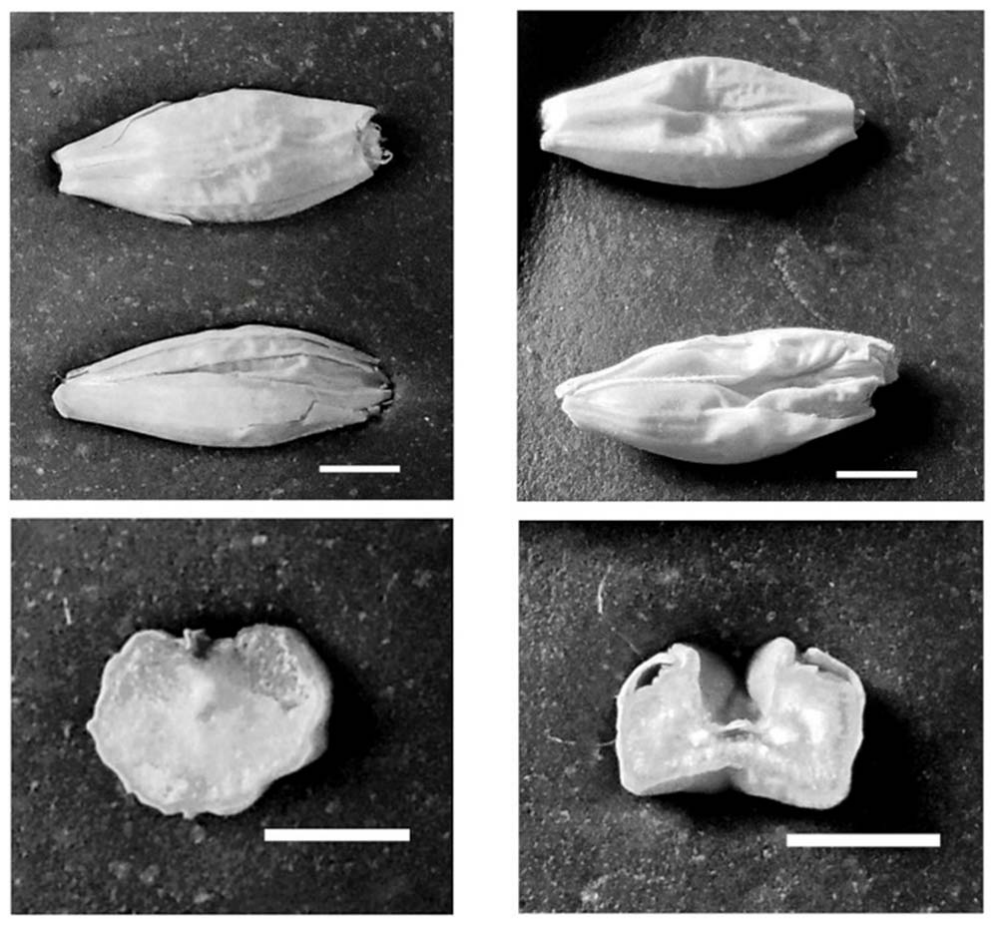
Seed morphology and transverse section of TILLING mutant line 1517-*SSIIa* (*Starch Synthase IIa*) (right) showing a shrunken phenotype, compared with cv. Morex wild type (left). From top to bottom: adaxial and abaxial seed views, and seed cross section. White bars  = 2 mm.

Molecular details about TILLING for five genes involved in starch metabolism were already reported in Bovina et al. [22] and will only be summarized here. TILLING was carried out in TILLMore, a TILLING population in the cultivar (cv.) Morex background which was chemically mutagenized using NaN_3_ [23]. The analyses identified an allelic series for each of the genes examined with a total number of 29 mutations [22]. Seeds of nine mutant lines carrying either missense or nonsense mutations in the five genes analyzed (*BMY1 GBSSI*, *LDA1*, *SSI* and *SSIIa*) were phenotypically characterized in this study (Table 1). Seeds of the mutant lines did not show any macroscopic differences in respect to Morex wild type (wt) with the exception of the line 1517-*SSIIa* (*Starch Synthase IIa*) that showed shrunken kernels with an empty cavity inside (Figure 1). These seeds were also lighter than wild-type ones (3.6±0.3 vs. 4.9±0.1 g/100-kernels).

### Total starch content

Total starch content was measured in whole flours obtained from two biological replicates for each of the nine mutant lines. The water content of the flours was very similar in all samples (≈9%, Table S1 in File S1). Morex wt contained 43% starch on a fresh weight basis, but this value was diminished by one third in mutant 1517-*SSIIa* (27%, P<0.01; Table 2). In no other mutants the total starch content was significantly different to the wild-type value (P<0.05, n = 2).

**Table 2:** Content of starch and amylose in seeds of TILLING mutant lines.

	% starch		% amylose (enzymatic)		% amylose (colorimetric)	
Morex	42.5±1.3	(100)	32.3±3.2	(100)	33.4	(100)
2253-*BMY1*	47.7±2.7	(112)	31.9±1.9	(99)	34.0	(102)
2682-*BMY1*	46.2±0.6	(109)	34.4±0.9	(107)	29.0	(87)
1090-*GBSSI*	38.9±0.8	(92)	9.5±3.1 *	(29)	8.8	(26)
905-*LDA1*	44.2±0.5	(104)	31.0±2.2	(96)	30.8	(92)
1132-*SSI*	41.8±2.4	(98)	32.6±2.8	(101)	36.1	(108)
1284-*SSI*	40.6±1.1	(96)	34.5±1.0	(107)	35.9	(107)
5850-*SSI*	42.1±2.3	(99)	31.0±1.4	(99)	36.7	(110)
1039-*SSIIa*	43.1±2.1	(101)	34.9±0.7	(108)	36.4	(109)
1517-*SSIIa*	26.5±0.1 **	(62)	47.5±5.0 *	(147)	48.9	(146)

Total starch content is expressed as percentage of fresh weight (water content in flours was about 9%, with no significant differences among samples, see Table S1 in File S1). Amylose was detected either by enzymatic or colorimetric methods and it is expressed as a percentage of total starch. To facilitate comparisons, all values are also reported in brackets as percentage of the corresponding wild type value. Total starch and relative amylose content was determined on two biological replicates. Significant differences were detected by Student's t-test (P<0.05 = *; P<0.01 = **). For comparison, colorimetric analysis of amylose was performed on a single biological sample for each genotype, and data shown are means of 3 technical replicates (standard deviations were in all cases below 10% of the mean value).

### Amylose content

Whole flours from two biological replicates were also analysed for amylose content by enzymatic assay. For comparison, the relative content of amylose was also determined colorimetrically with similar results (Table 2). Wild type starch contained 32% amylose and similar values were detected in seven over nine mutants (amylose/amylopectin ratio 0.47). However, mutant 1090-*GBSSI,* carrying a missense mutation in *Granule Bound Starch Synthase I*, contained only one third of the normal amylose content in its grain starch (9%, P<0.05; amylose/amylopectin ratio 0.10), and mutant 1517-*SSIIa,* carrying a nonsense mutation in *Starch Synthase IIa*, contained much more amylose than the wild type (47%, P<0.05; amylose/amylopectin ratio 0.88; Table 2).

### SDS-PAGE analysis

**Figure 2:**
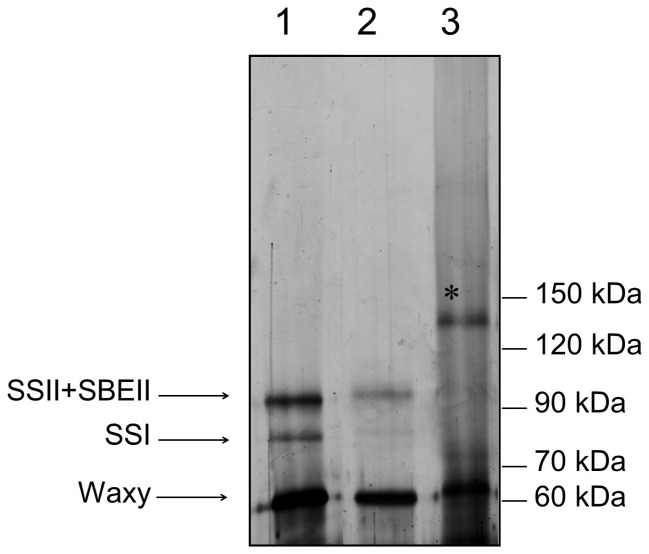
Electrophoretic separation (SDS–PAGE) of starch granule proteins extract from barley wild type cv. Morex (1) and barley mutants 1284-*SSI* (2) and 1517-*SSIIa* (3). The bands corresponding to starch synthase II and starch branching enzyme II (SSII+SBEII), starch synthase I (SSI) and granule-bound starch synthase (GBSSI) are indicated. In lane 3, the high molecular weight band marked with an asterisk is probably due to impurities present in the starch preparation obtained from the shrunken seeds of line 1517-*SSIIa*. Molecular weight standard is schematically reported on the right.

In order to assess whether the nine mutations identified had an effect on the electrophoretic protein profile typical of the starch granule proteins of barley, SDS-PAGE analysis was performed. With the exception of the lines 1517-*SSIIa* and 1284-*SSI*, all the mutants showed a profile identical to Morex wt, characterized by three major bands corresponding to SSIIa (overlapped with Starch Branching Enzyme II, SBEII), SSI and GBSSI [29] (Figure S1 in File S1). The absence of the SSIIa enzyme was confirmed in the line 1517-*SSIIa*. Notably this mutant appeared to lack also SBEII and SSI isoforms. Moreover, although mutant 1284-*SSI* has no premature stop codon in the *SSI* gene, a drastic reduction of the SSI band was observed in starch granules (Figure 2).

### Starch granules morphology

**Figure 3:**
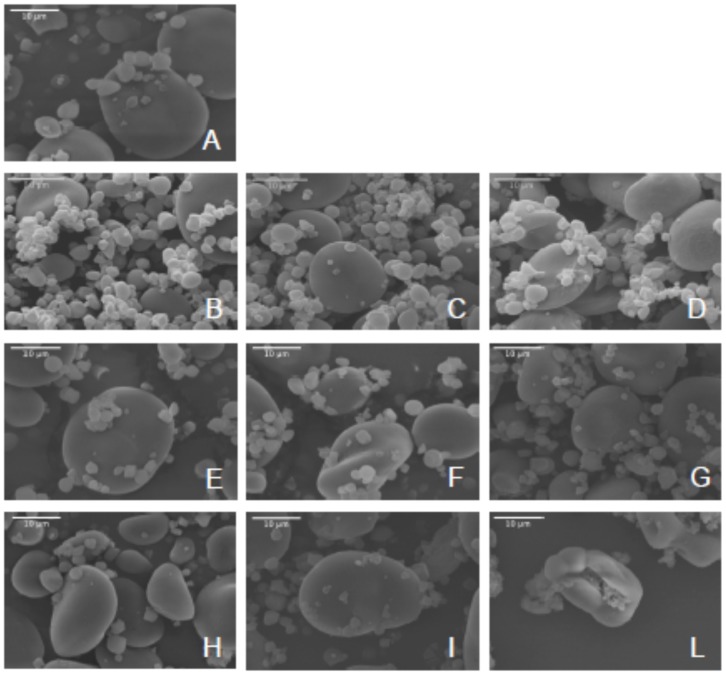
Scanning Electron Microscopy (SEM) analysis of starch granules from barley cv. Morex wild-type (A) and mutants 2253-*BMY1* (B), 2682-*BMY1* (C), 1090-*GBSSI* (D), 905-*LDA1* (E), 1132-*SSI* (F), 1284-*SSI* (G), 5850-*SSI* (H), 1039-*SSIIa* (I), 1517-*SSIIa* (L). Scale bar: 10 µm.

Starch extracts from wild-type and mutant grains were analysed by Scanning Electron Microscopy (SEM). With the exception of 1517-*SSIIa*, starch granules of all remaining samples were quite regularly shaped (Figure 3 and Figure S2 in File S1). A quantitative analysis of granules dimensions was performed by collecting the length of the major and minor axis of 200–500 granules for each biological sample from their SEM digital images. Distribution of granule dimensions was clearly bimodal in all samples (Figure S3 in File S1), with a major sub-population of small spherical granules (major axis <8 µm, B-granules), and a minor sub-population of larger discoid particles with a major axis varying between 8 and 30 µm (A-granules). Distributions based on minor axis were qualitatively identical to those based on the major axis (not shown). Differently from all other mutants, starch of 1517-*SSIIa* contained irregularly shaped A-granules typically appearing like deflated spheres (Figure 3 and Figure S2 in File S1). B-granules were also irregular in shape and agglomerated. These features prevented a quantitative determination of A and B-type particles in 1517-*SSIIa* samples.

**Figure 4:**
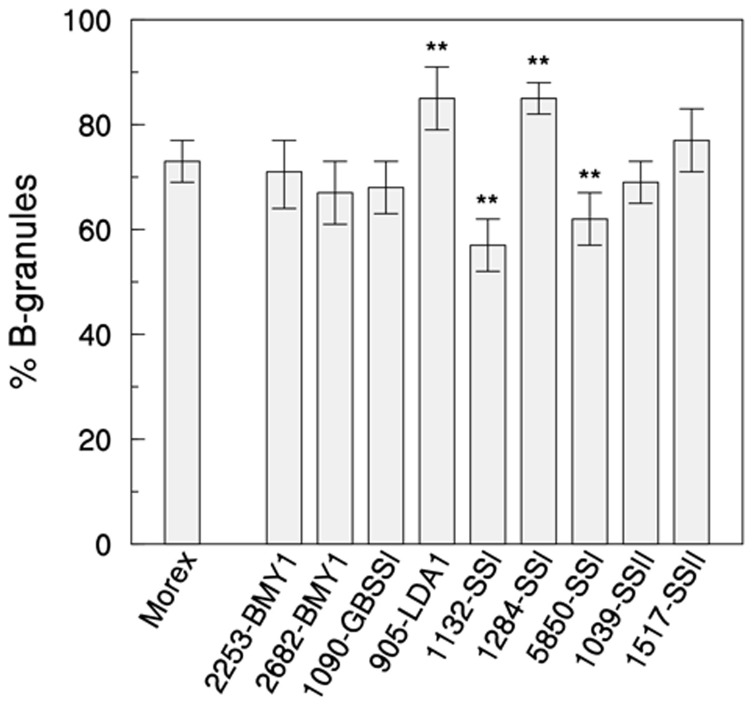
Percentage of B-type granules (diameter <8 µm) in grain starch of barley wild-type cv. Morex and mutant lines. Granules size distribution was determined on 10 couples of SEM images randomly collected for each genotype. Data shown are means ±SD (n = 10). Statistically significant differences between mutants and wild type mean values were estimated by Student's t-test (P<0.01) and are highlighted by a double asterisk (**).

The percentage of B-granules (<8 µm) in wild-type purified starch was 73% (SD). A similar value was found in mutants of *BMY1*, *GBSSI* and *SSIIa* (Figure 4). In the four remaining mutants the percentage of B-granules differed significantly from the wild type Morex (P<0.01). B-granules were less abundant in two soluble starch synthase mutants, 1132-*SSI* (57%) and 5850-*SSI* (62%), but relatively more abundant in mutant 1284-*SSI* of the same gene (85%) and in mutant 905-*LDA1* of limit dextrinase I (85%) (Figure 4). All these four mutants contained missense mutations (Table 1).

The average size of A- and B-granules in each sample was also analysed. In two mutants (5850-*SSI* and 1039-*SSIIa*, both missense mutations), A-type granules were significantly smaller (−25% major axis) than wt ones (17.1 µm) (Table 3; P<0.01). On the other hand, A-granules of 1284-*SSI* mutant were significantly larger (+15%) than wt ones. The average diameter of B-particles varied between 2.3 and 3.5 µm in all samples, with no significant differences between wt and mutants (Table 3). Interestingly, two missense mutants of *SSI* displayed symmetrical properties in A-granules size and frequency: in mutant 1284-*SSI* A-granules were larger but less abundant, while in mutant 5850-*SSI* they were relatively more numerous, but smaller in size (Table 3 and Figure 4).

**Table 3:** Length of major axis in A-type and B-type starch granules.

	B-granules, major axis [µm]	A-granules, major axis [µm]
Morex	3.01±1.23	17.08±4.88
2253-*BMY1*	2.50±0.77	17.97±5.17
2682-*BMY1*	2.80±1.19	18.18±5.47
1090-*GBSSI*	2.26±0.86	16.49±7.24
905-*LDA1*	2.83±1.04	16.68±5.10
1132-*SSI*	3.45±1.09	17.70±4.38
1284-*SSI*	3.02±1.03	19.69±4.84 **
5850-*SSI*	3.38±1.86	13.15±3.41 **
1039-*SSIIa*	3.27±1.74	12.92±3.39 **
1517-*SSIIa*	Nd	Nd

Data are means ±SD of 200–500 granules measured for each genotype. Statistically significant differences as determined by Student's t-test are indicated (P<0.01 = **). Nd, not determined (starch granules in 1517-*SSIIa* mutant were irregular in shape).

### Crystallinity of starch granules

**Figure 5:**
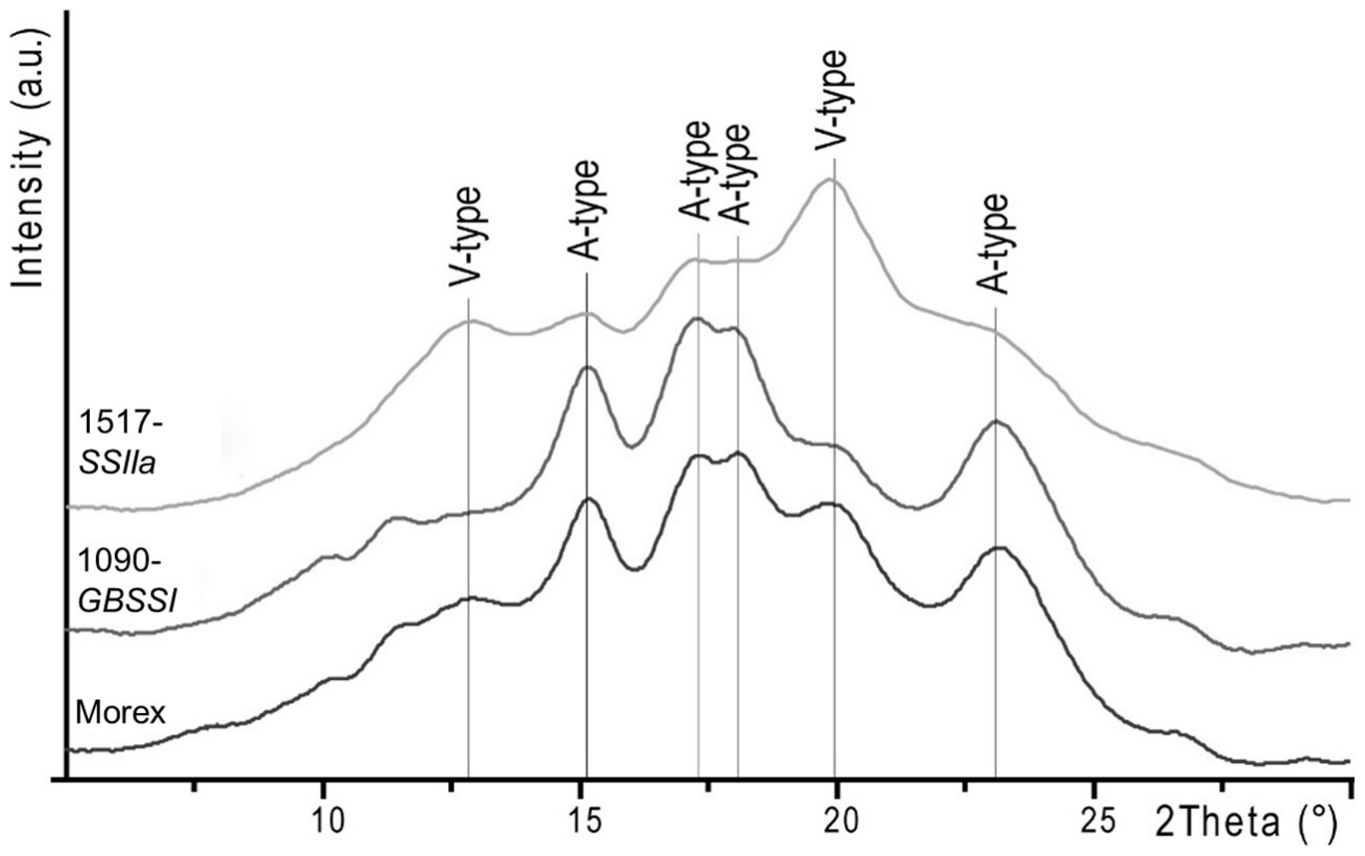
X-ray diffraction patterns of native starch extracts from barley wild type cv. Morex (black) and mutants 1090-*GBSSI* (grey) and 1517*-SSIIa* (light grey). The characteristic peaks of the A-type and V-type polymorphs are indicated.

Crystallinity of starch granules was evaluated by X-ray powder diffraction. The crystallinity of wild-type starch was estimated as 29% and this value ranged between 26 and 33% in all mutants (Table 4), with no clear correlation between the degree of crystallinity and other phenotypic characters previously recorded. On the contrary, the type of crystallinity, as detected from the X-ray diffraction patterns, was more variable. In wild-type starch we estimated a large predominance of the A-type crystal pattern (81%), with a minor contribution of the V-type (Figure 5). No evidence for B-type crystallinity was obtained from diffraction patterns of wild type and mutants. In most of the mutants, the type of crystallinity was similar to that observed in wild type starch, *i.e.* 78–83% A-type and 17–22% V-type. Interestingly, however, in the low-amylose 1090-*GBSSI* mutant, crystallinity was almost exclusively of the A-type (92%) whereas in the high amylose 1517-*SSIIa* mutant crystallinity was prevalently of the V-type (76%) (Figure 5 and Table 4).

**Table 4:** Percentage crystallinity and relative intensity of diffraction peaks at 15.1° and 19.7°.

	% crystallinity	I_A-type_	I_V-type_
Morex	0.29	0.81	0.19
*2253-BMY1*	0.30	0.82	0.18
2682-*BMY1*	0.33	0.80	0.20
1090-*GBSSI*	0.26	0.92	0.08
905-*LDA1*	0.27	0.81	0.19
1132-*SSI*	0.28	0.79	0.21
1284-*SSI*	0.27	0.79	0.21
5850-*SSI*	0.27	0.80	0.20
1039-*SSIIa*	0.26	0.78	0.22
1517-*SSIIa*	0.29	0.24	0.76

These latter data represent a relative estimation of A-type and V-type crystallinity, respectively.

## Discussion

Starch structure and chemical composition are genetically determined by a large set of genes [5], [6], [8], [30] and the potential for obtaining different types of starch by screening natural or induced genetic variability is huge. TILLING provides a non-transgenic approach to explore this potential [7], [12], [22], [31], [32], [33], [34], [35], [36]. We exploited TILLING to identify and phenotypically characterize new alleles of five genes involved in grain starch metabolism of barley.

### Granule bound starch synthase (*GBSSI*)

Granule-bound starch synthase I (*GBSSI*) [4] is specifically expressed in the endosperm of barley [30] where it is known to exert a tight control on the biosynthesis of amylose [37]. GBSSI is coded by the *waxy* locus and barley cultivars with altered GBSSI activity contain altered levels of amylose in grains, ranging between 0 and 41% of total starch [29]. Besides amylose, GBSSI is also involved in the synthesis of extra long glucan chains of amylopectin, such that also amylopectin may be modified in *waxy* mutants [38], [39], [40]. Low-amylose varieties can be used for food applications because of their peculiar starch features (low gelatinization temperature and retrogradation), that confer high freeze-thaw stability and anti-stailing properties to processed food [41], [42].

Here we report a new allele of *GBSSI* with a G493E point mutation. Grain starch of this mutant (1090-*GBSSI*) contains less than 10% amylose (vs. 30% of wild-type) and is thus defined low-amylose or near-waxy. Crystallinity of 1090-*GBSSI* starch was found to be largely A-type, with a minor contribution of the V-type pattern. A similar profile has been previously reported in low amylose barley [43], [44].

**Figure 6:**
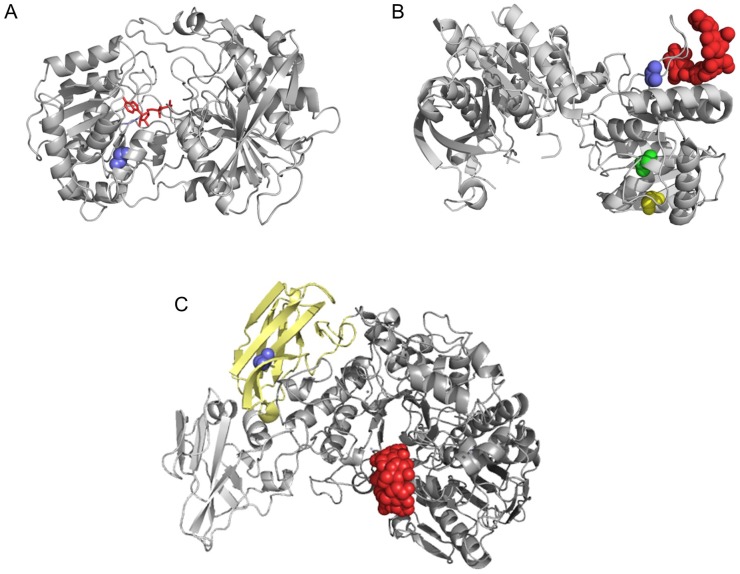
Localization of point mutations in the 3D structures of barley GBSSI, SSI and LDA1. **A) Barley GBSSI was modelled by Swissmodel using the catalytic domain of wild-type rice GBSSI complexed with ADP as a template (pdb 3VUF).** The two mature proteins are 84% identical in sequence. The main chain of glycine-493 is represented by blue spheres. In mutant 1090-*GBSSI,* glycine-493 is substituted by a glutamate (G493E). Co-crystallized ADP of the rice GBSSI structure (3VUF) is superimposed to highlight the adenine nucleotide binding site. B) Crystal structure of barley SSI, co-crystallized with a molecule of maltopentaose (red spheres) (pdb 4HLN). Main chain atoms of mutated residues are represented by coloured spheres: blue (G576D in mutant 5850-*SSI*), green (T522I in mutant 1132-*SSI*) and yellow (G509E in mutant 1284-*SSI*). C) Crystal structure of barley LDA1 (pdb 2X4B). The carbohydrate binding module CBM48 is coloured yellow. Residue no. 270 (blue spheres corresponding to main chain atoms) is part of the CBM48 domain. Mutant 905-*LDA1* carries a V270I mutation. A molecule of betacyclodextrine (red spheres) co-crystallized with the protein highlights the putative active site.

Plant starch synthases (both granule bound and soluble isoforms) are proteins of about 60–120 kDa that belong to the glycosyltransferase family GT-5 [4]. They typically contain a catalytic domain formed by two Rossman fold domains delimiting a deep cleft where the catalytic site is located (Figure 6). In plant starch synthases, the catalytic domain is often preceded by an N-terminal sequence of variable length and no clear function. The crystal structure of the catalytic domain of rice GBSSI [45] was used as a template to model barley GBSSI (the two proteins are 84% identical in amino acid sequence). According to the model, glycine-493 belongs to an alpha-helix of the second, C-terminal Rossman fold domain at approximately 10 Å from the ADP binding pocket [45] and 6 Å from the conserved STGGL motif suspected to be involved in catalysis and/or substrate binding [29].

Several near-waxy cultivars are known in barley, all carrying a large deletion in the promoter region of the *GBSSI* gene that results in strongly diminished expression of the enzyme [29], [46]. Waxy cultivars with no detectable amylose are also known (e.g. cv. CDC Alamo and CDC Fibar) but they contain point mutations in the coding sequence that likely cause complete inactivation of the enzyme without drastically affecting the protein abundance in starch granules [29]. Interestingly, mutation G493E in 1090-*GBSSI* seems to modulate, rather than inactivate, enzyme activity as demonstrated by the residual content of amylose (10%) detected in its starch. Moreover, this effect is obtained without altering protein expression, as suggested by the SDS-PAGE pattern identical to the wild-type. Indeed, mutation G493E may prove useful for the understanding the little known catalytic mechanism of GBSSI.

### Limit dextrinase (*LDA1*)

Together with isoamylases (ISAs), limit dextrinases (LDAs, also known as pullulanases) constitute the set of starch debranching enzymes. The role of ISAs in removing excess branches of amylopectin formed by branching enzymes is well known [8]. In the absence of isoamylases, starch is synthesized in a highly branched form known as phytoglycogen [47]. Barley limit dextrinase is coded by a single gene (*LDA1*) and is apparently involved both in starch biosynthesis and degradation [48]. *In vivo*, the activity of LDA1 is regulated by a proteinaceous inhibitor LDI [49], and antisense transgenic barley with lower expression of LDI showed higher LDA1 activity and a lower percentage of B-granules (*i.e.* inhibition of granule initiation) and lower amylose/amylopectin ratio [50]. It was suggested that LDA, which is expressed when B-granules are formed, may play a role in reducing the amount of primers that allows the nucleation of small B-granules. In our study, we have found a mutant of *LDA1* (905-*LDA1*) with a higher percentage of small B-granules that further supports the role of this enzyme in starch granule initiation.

The tridimensional structure of barley *LDA1* has been solved [51]. The protein is made of four domains: the N-terminal domain, the CBM48 domain, the catalytic domain and the C-terminal domain. Valine-270 of *LDA1*, that in mutant 905-*LDA1* is substituted by a more bulky isoleucine, belongs to the carbohydrate binding module CBM48 and is located close to the interface with the catalytic domain (Figure 6). It is plausible that the substitution of valine in isoleucine (V270I) in the CBM48 domain may reduce the capability of the protein to bind glucans and in turn inhibit, albeit indirectly, its scavenging activity toward primers of granules nucleation. Consistently, starch of mutant 905-*LDA1* is formed by a larger number of granules (predominantly small B-granules) and the role of LDA1 in controlling granule nucleation is further supported.

### Soluble starch synthase I (*SSI*)

In plants, soluble starch synthases are divided into four classes with different specificity (from *SSI* to *SSIV*), and some of them are represented by more than one isoform [4], [8], [9]. While *SSIV* is probably involved in starch granule initiation [52], *SSI* preferentially synthesize short glucan chains using short amylopectin chains as substrate [5], [8]. These short amylopectin chains may then be prolonged by *SSII* and *SSIII* [5], [8], but individual roles and cooperation between different starch synthases in building the starch granule are still undefined, in barley at least. Mutants in rice and wheat clearly suggest that the activity of the different starch synthases is not necessarily sequential and the lack of SSI may be partially compensated *in vivo* [8], [53], [54].

In cereals *SSI* is represented by a single isoform. We have analysed three missense mutations in *SSI* and all of them showed starch phenotypes consisting in modifications in either size or frequency of A- and B-granules. However, our results may appear contradictory: in fact two mutants showed a higher % of A-type granules (1132-*SSI* and 5850-*SSI*), while the third mutant had more B-type granules (1284-*SSI*). Morever, the A-granules of mutant 5850-*SSI* were more abundant but smaller in size and, symmetrically, the A-granules of mutant 1284-*SSI* were larger but less frequent. Only in mutant 1132-*SSI* the higher percentage of A-granules was not compensated by a reduction in size. The unexpected phenotypic difference between the three mutant lines may be due to currently unknown additional background mutations present in the genome of TILLMore mutant lines [23].

However, some hints could be obtained from the recently solved crystallographic structure of barley *SSI* [55] (Figure 6). The G576D substitution of mutant 5850-*SSI* is localized at the base of a loop involved in the formation of a high affinity binding site for maltooligosaccharides. This site is 30 Å away from the putative catalytic site, but is believed essential for colocalizing branched glucans and *SSI*, thereby favoring catalysis. Moreover, the starch phenotype associated to the G576D mutation (smaller A-granules) may suggest a role of this site in controlling the final size of large starch granules. The other two point mutations here described (Figure 6) are localized in regions of the protein not yet characterized, providing no suggestions to understand their role. Nevertheless, the high percentage of large A-granules in mutant 1132-*SSI* is interesting because this is a desirable trait for malting [10].

Initiation of A and B-granules are separated events, although little is known of the genetic control of this trait in Triticeae. In barley, A-granules are nucleated at 4–14 days post-anthesis, during endosperm cell division, while small B-granules are nucleated later, during endosperm cell growth [56]. A QTL controlling B-granules initiation was recently described in wild wheat Aegilops [10] and in Arabidopsis, SSIV is believed to positively regulate granule initiation [52]. Recently the suppression of *SSI* expression in wheat grains using RNAi technology led to the production of lines with a reduced proportion of B-granules [54]. All three *SSI* barley mutants analysed in this work showed an abnormal distribution between large and small granules. However, because of the complexity of our results, any conclusion about a possible role of SSI in granule initiation in barley is premature.

### Soluble starch synthase IIa (*SSIIa*)

*SSIIa* is the major SS isoform of barley endosperm during grain filling [4]. Mutant *sex6* of barley cv. Himalaya has no *SSIIa* activity and produces shrunken kernels containing starch made of up to 70% amylose [15], [16]. *SSIIa* knock-out mutants are particularly interesting for industrial applications because of their higher level of amylose and resistant starch in the endosperm. Resistant Starch is associated with several human health benefits, including the prevention of the colon cancer, type-II diabetes and obesity [13], [19]. In this work, we identified a mutant line with A-granule of smaller size carrying a missense mutation (1039-*SSIIa*), and a *SSIIa* null mutant (1517-*SSIIa*) characterized by small/shrunken seeds and containing less starch with more amylose (48% of grain starch is made of amylose in this mutant). The SDS-PAGE analysis of starch extracted from 1517-*SSIIa* confirmed the absence of the protein *SSIIa*, together with *SBEII* and *SSI* isoforms. The simultaneous absence of *SSIIa*, *SBEII* and *SSI* in the starch granule was already observed in *SSIIa* mutants of barley, bread and durum wheat [14], [57], [58].

Starch crystallinity of 1517-*SSIIa* was largely characterized by a V-type diffraction pattern suggesting the formation of lipid-amylose complexes, similarly to those observed in the *sex6* mutant [15]. In the missense mutant 1039-*SSIIa*, in spite of the smaller size of A-granules, no other starch parameters including crystallinity were significantly affected. In barley endosperm, *SSIIa* was shown to extend short amylopectin glucan chains of 3–8 glucose units to chains of up to 35 units [15]. Consistently, the lack of *SSIIa* negatively affects amylopectin synthesis, more than amylose synthesis [16], and mutant 1517-*SSIIa* is fully consistent with these results.

## Conclusions

TILLING of five genes encoding enzymes involved in starch metabolism enabled us to identify seven new alleles that are associated with new starch phenotypes in terms of amylose/amylopectin ratio, or crystal packing, or distribution of A- and B-granules, or size of A-granules (Table 1). Our results confirmed the role played by granule-bound starch synthase (*GBSSI*) in controlling amylose biosynthesis and, conversely, the role played by soluble starch synthase IIa (*SSIIa*) in controlling amylopectin synthesis. Starch granule initiation appeared to be controlled by limit dextrinase (*LDA1*), and size of A-granules by starch synthases IIa. Thanks to their physical-chemical properties, these new alleles deserve further attention in order to investigate their possible interest in nutritional uses or industrial applications.

## Supporting Information

File S1 Figure S1, SDS–PAGE separation of starch protein extract from cv. Morex and barley mutants. Figure S2, SEM analysis of starch granules from cv. Morex (A) and barely mutants 2253-*BMY1* (B), 2682-*BMY1* (C), 1090-*GBSSI* (D), 905-*LDA1* (E), 1132-*SSI* (F), 1284-*SSI* (G), 5850-*SSI* (H), 1039-*SSIIa* (I), 1517-*SSIIa* (L). Scale bars: 10, 20, and 30 µm. Table S1, Water content in whole flours of TILLING mutant lines. Dry weight was obtained after incubation of samples at 80°C for 24 h. Data are means ±SD (n = 4).
(PDF)
