# Supplementary material for: New Starch Phenotypes Produced by TILLING in Barley
Source: PLoS One. 2014 Oct 1;9(10):e107779. doi: 10.1371/journal.pone.0107779 (PMC4182681; doi:10.1371/journal.pone.0107779)
Supplement: File S1 — Figure S1, SDS–PAGE separation of starch protein extract from cv. Morex and barley mutants. Figure S2, SEM analysis of starch granules from cv. Morex (A) and barely mutants 2253-BMY1 (B), 2682-BMY1 (C), 1090-GBSSI (D), 905-LDA1 (E), 1132-SSI (F), 1284-SSI (G), 5850-SSI (H), 1039-SSIIa (I), 1517-SSIIa (L). Scale bars: 10, 20, and 30 µm. Table S1, Water content in whole flours of TILLING mutant lines. Dry weight was obtained after incubation of samples at 80°C for 24 h. Data are means ±SD (n = 4). (PDF) [file pone.0107779.s001.pdf]

	Water content (%)
Morex	8.78±1.10
2253-*BMY1*	9.16±1.48
2682-*BMY1*	7.59±1.63
1090-*GBSSI*	9.28±1.98
905-*LDA1*	9.69±1.20
1132-*SSI*	9.74±0.26
1284-*SSI*	10.26±1.44
5850-*SSI*	9.77±0.61
1039-*SSIIa*	9.97±1.55
1517-*SSIIa*	8.58±1.64

Table S1 – Water content in whole flours of TILLING mutant lines. Dry weight was obtained after incubation of samples at 80°C for 24h. Data are means±SD (*n*=4).

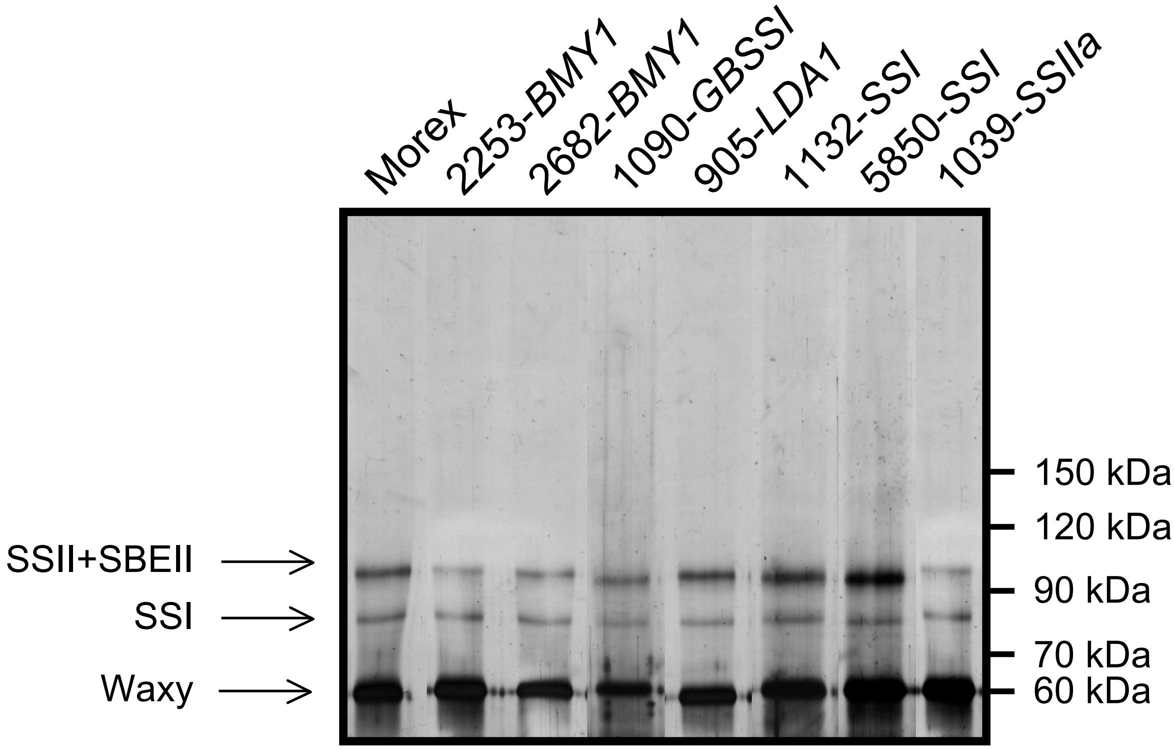

Figure S1 - SDS–PAGE separation of starch protein extract from cv. Morex and barley mutants.

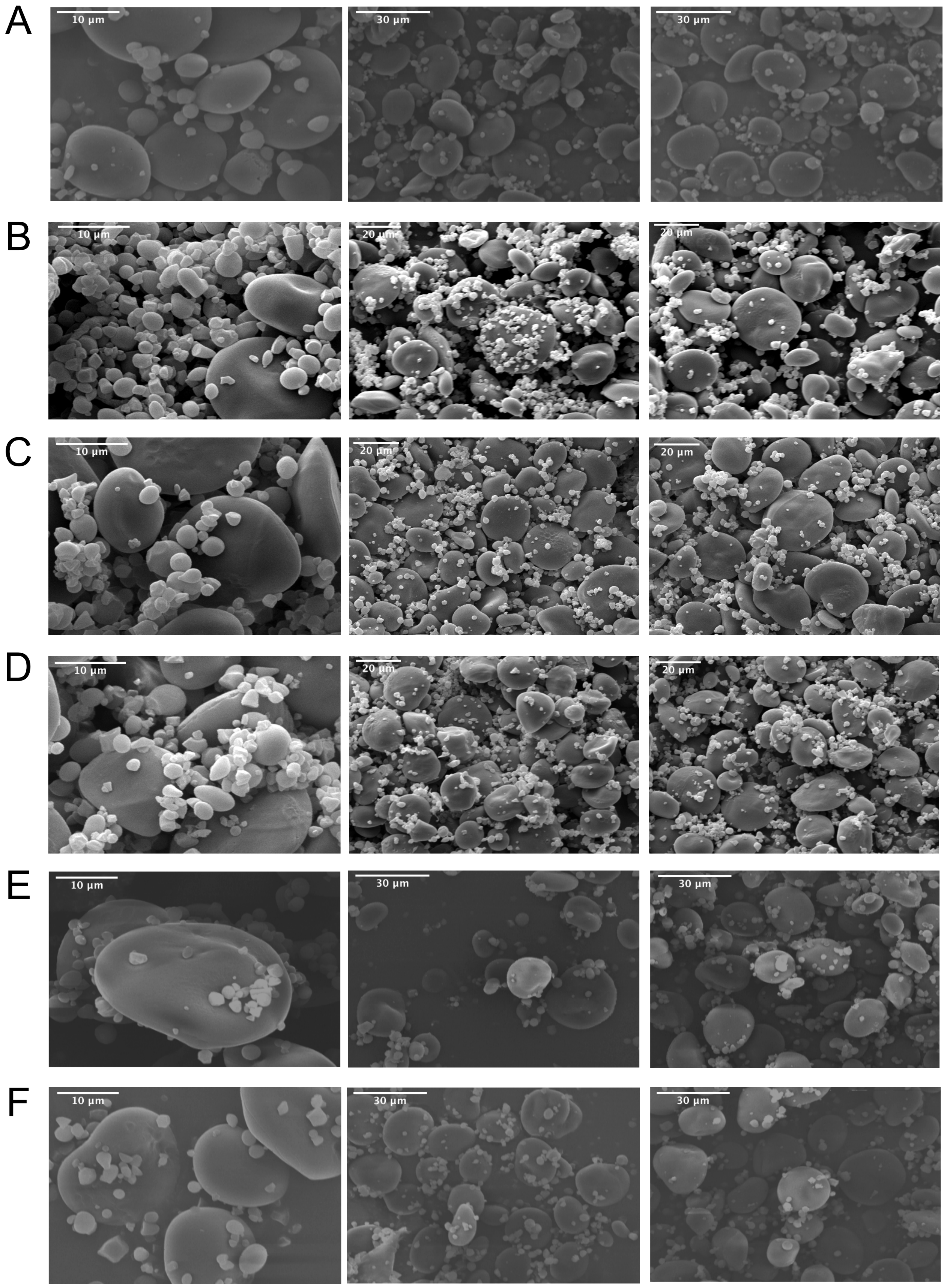

A
10 µm
30 µm
30 µm
B
10 µm
20 µm
20 µm
C
10 µm
20 µm
20 µm
D
10 µm
20 µm
20 µm
E
10 µm
30 µm
30 µm
F
10 µm
30 µm
30 µm

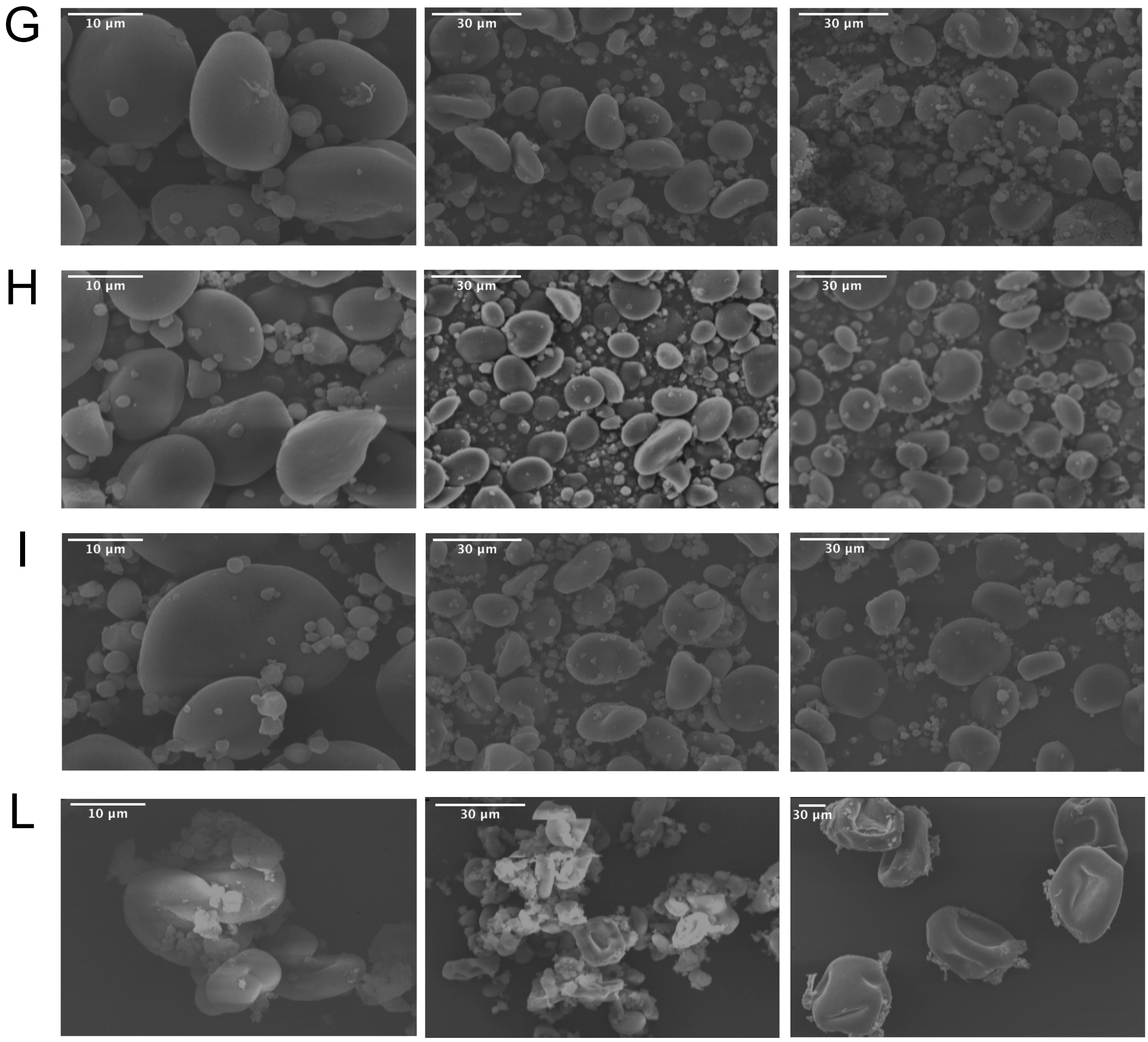

Figure S2 - SEM analysis of starch granules from cv. Morex (A) and barely mutants 2253-*BMY1*(B), 2682-*BMY1* (C), 1090-*GBSSI* (D), 905-*LDA1* (E), 1132-*SSI* (F), 1284-*SSI* (G), 5850-*SSI* (H), 1039-*SSIIa* (I), 1517-*SSIIa* (L). Scale bars: 10, 20, and 30 µm.
